# Identification of miR-27b as a Novel Signature from the mRNA Profiles of Adipose-Derived Mesenchymal Stem Cells Involved in the Tolerogenic Response

**DOI:** 10.1371/journal.pone.0060492

**Published:** 2013-04-16

**Authors:** Kuang-Den Chen, Shigeru Goto, Li-Wen Hsu, Tzu-Yang Lin, Toshiaki Nakano, Chia-Yun Lai, Yen-Chen Chang, Wei-Teng Weng, Yur-Ren Kuo, Chih-Chi Wang, Yu-Fan Cheng, Yen-Ying Ma, Chih-Che Lin, Chao-Long Chen

**Affiliations:** 1 Center for Translational Research in Biomedical Sciences, Liver Transplantation Program and Departments of Surgery, Kaohsiung Chang Gung Memorial Hospital and Chang Gung University College of Medicine, Kaohsiung, Taiwan; 2 Iwao Hospital, Yufuin, Japan; 3 Department of Chemistry, National Cheng Kung University, Tainan, Taiwan; 4 Graduate Institute of Clinical Medical Sciences, Kaohsiung Chang Gung Memorial Hospital and Chang Gung University College of Medicine, Kaohsiung, Taiwan; 5 Department of Plastic and Reconstructive Surgery, Kaohsiung Chang Gung Memorial Hospital and Chang Gung University College of Medicine, Kaohsiung, Taiwan; 6 Department of Diagnostic Radiology, Kaohsiung Chang Gung Memorial Hospital and Chang Gung University College of Medicine, Kaohsiung, Taiwan; 7 Department of Obstetrics and Gynecology, Kaohsiung Chang Gung Memorial Hospital and Chang Gung University College of Medicine, Kaohsiung, Taiwan; University of Medicine and Dentistry of New Jersey, United States of America

## Abstract

Adipose-derived mesenchymal stem cells (adipose-derived MSCs, ASCs) possess the ability to differentiate into multiple tissue types and have immune-modulatory properties similar to those of MSCs from other origins. However, the regulation of the MSC-elicited immune-modulatory activity by specific microRNA (miRNA) mechanisms remains unexplored. Gene expression profiling with knowledge-based functional enrichment analysis is an appropriate approach for unraveling these mechanisms. This tool can be used to examine the transcripts and miRNA regulators that differentiate the rat tolerogenic orthotopic liver transplantation (OLT; DA liver into PVG) and rejection OLT (DA liver into LEW) models. In both models, the rejection reaction was observed on postoperative day 7∼14 (rejection phase) but was overcome only by the PVG recipients. Thus, the global gene expression patterns of ASCs from spontaneously tolerant (PVG) and acute rejecting (LEW) rats in response to LPS activation were compared. In this study, we performed miRNA enrichment analysis based on the analysis of pathway, gene ontology (GO) terms and transcription factor binding site (TFBS) motif annotations. We found that the top candidate, miR-27, was specifically enriched and had the highest predicted frequency. We also identified a greater than 3-fold increase of miR-27b expression in the ASCs of tolerant recipients (DA to PVG) compared to those of rejecting recipients (DA to LEW) during the rejection phase in the rat OLT model. Furthermore, our data showed that miR-27b knockdown has a positive influence on the allosuppressive activity that inhibits T-cell proliferation. We found that miR-27 knockdown significantly induced the expression of CXCL12 in cultured ASCs and the expression of CXCL12 was responsible for the miR-27b antagomir-mediated inhibition of T-cell proliferation. These results, which through a series of comprehensive miRNA enrichment analyses, might be relevant for stem cell-based therapeutic applications in immunosuppressive function using ASCs.

## Introduction

Mesenchymal stem cells (MSCs) are resident mesoderm-derived stromal cells from the bone marrow, peripheral blood, and adipose tissue. MSCs are defined as adherent, fibroblastoid-like cells with the capacity to differentiate into mesenchymal and non-mesenchymal cell lineages [1]. In addition to their potential for clinical applications in tissue repair, bone marrow-derived MSCs (BM-MSCs) are potent immune modulators that are involved in various immune disorders [1]–[5]. Because of their high accessibility, MSCs isolated from liposuctioned fat tissues (adipose-derived MSCs or ASCs) have emerged as an attractive resource for cell therapy. ASCs have also been reported to inhibit the activation, proliferation, and function of immune cells, including T cells, B cells, NK cells, and antigen-presenting cells (APCs) [5], [6]. Because of their biological properties, such as their ability to undergo differentiation and mediate immunosuppression, ASCs constitute an interesting cell population to consider for cell therapy and regeneration treatment.

MicroRNAs (miRNAs) are a species of single-stranded small non-coding RNAs that are 21–23 nucleotides in length. They exert their effects by annealing to complementary sites in the 3′UTR of target mRNAs. miRNAs inhibit de novo protein synthesis of the target mRNA by repressing the translation of the transcript or by accelerating transcript breakdown. They regulate the expression of the majority of protein-coding transcripts. Many cellular processes, including proliferation, differentiation, apoptosis, and hematopoiesis are regulated by miRNAs [7]–[9]. Recent evidence indicates that miRNAs are pivotal in controlling immune responses and suggests that abnormalities in miRNAs are associated with diseases [10]. Moreover, several miRNAs have recently been shown to downregulate Toll-like receptor (TLR) signaling. This signaling pathway plays an important role in innate immunity through the recognition of pathogenic molecules and also mediates the recruitment of the adaptive immune response [11], [12]. However, the current knowledge of the miRNAs involved in the immuno-modulatory abilities of the MSCs is limited. Therefore, exploring putative miRNA regulators and the role of miRNAs in the immunosuppressive activity of MSCs may help identify novel targets to augment the therapeutic potential of these cells after transplantation. The purpose of this study was to identify the transcripts and specific pathways that are differentially expressed between the ASCs isolated from naïve recipient rats with acute rejection (LEW) and spontaneous tolerance (PVG). We determined the transcriptional profiles of these two populations of cells with and without LPS stimulation. Pathway- and gene ontology (GO)-based enrichment analyses and combinatorial analysis with previously curated miRNA annotations were applied to the transcriptional data. Further, we aimed to gain insight into the global regulatory organization of transcriptional networks differentially activated in the ASCs of the LEW and PVG rats. To achieve this goal, we further screened *cis*-regulatory promoter motifs computationally using the PRIMA algorithm to reveal the transcription factors (TFs) that were differentially expressed between the conditions [13]. We also identified GO terms that were significantly enriched in the identified TFs to validate the *in silico* consistency of associated functions.

## Results

### Differentially expressed transcripts in the ASCs

**Figure 1:**
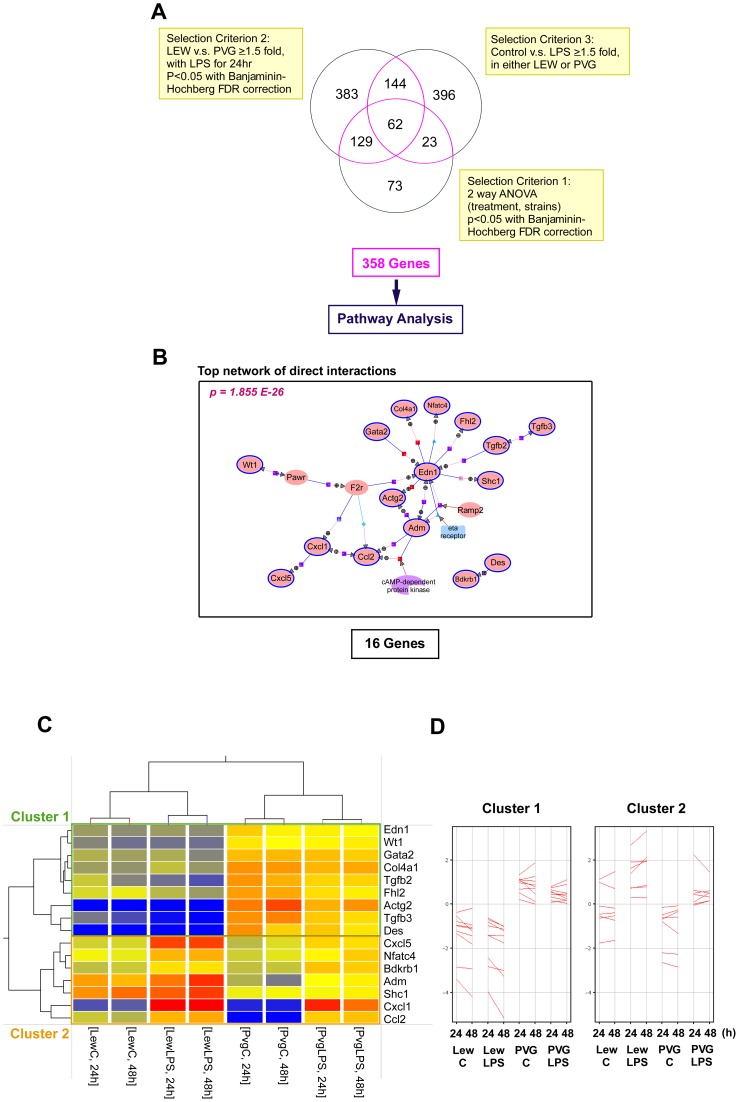
Overlapping differentially expressed genes identified by comparisons between the ASCs of the LEW and PVG groups and the top interacting network among the shared genes identified by the 3 comparisons. (A) A Venn diagram detailing the number of differentially expressed genes that were shared and distinct between three comparisons (List 1: comparing expression in the LEW-LPS group at 24 h and the PVG-LPS group at 24 h. Genes with greater than a 1.5-fold change were identified with the Mann-Whitney unpaired test(p<0.05). List 2: comparing the LEW and PVG groups with or without LPS stimulation at 24 h. Genes with greater than a 1.5-fold change at least 1 condition were considered. List 3: results of the 2-way ANOVA with Benjamini-Hochberg multiple testing correction (p<0.05). The number of transcripts within each subset is highlighted in the yellow boxes adjacent to the Venn diagram. The number of genes shared by at least 2 of the 3 gene lists is indicated by the purple color. The 358 total genes were subjected to further network analysis. (B) The functional interaction networks among the 358 genes were analyzed using the significant pathway analysis tool in Genespring GX. The top networks of significant “direct interactions” and their associated p-values are shown. (C) Complete hierarchical linkage analysis was applied to the 16 genes identified in the top network. Each column represents an individual pooled sample, and each row represents a specific gene. Red indicates high relative expression, and blue indicates low relative expression. The upper panel depicts 9 genes that are responsible for low expression levels in the ASCs of the LEW rats (cluster 1). The lower panel depicts 7 genes that are differentially expressed after LPS treatment. The relative gene expression levels of the 16 genes are also presented (D).

Messenger RNA was isolated from the ASCs of naïve LEW and PVG rats with and without LPS treatment for 24 h or 48 h. The samples were prepared for hybridization to microarrays and analyzed as described in the Materials and Methods. This approach allowed us to examine the differences in ASC function between the LEW and PVG rats. All microarrays displayed high signal to noise ratios. Overall, 20177 of the total of 22523 probes (89.6%) showed at least 1 P (present) or M (marginal) flag compared to background. From the 20177 probes 287 transcripts were found to be differentially expressed between naïve LEW and naïve PVG ASCs with and without different durations of LPS treatment using 2-way ANOVA with a false discovery rate below 5% of Benjamini-Hochberg correction. Furthermore, 718 differentially expressed transcripts were identified using another statistical criterion, each with a ≥1.5 fold change between the LEW and PVG groups treated with LPS for 24 h. The Mann-Whitney unpaired test with the Benjamini-Hochberg multiple testing correction and a statistical threshold of p-value <0.05 was applied, and 191 out of the 718 transcripts intersected with those found in the 2-way ANOVA. Moreover, the results of 625 transcripts for differential expression with and without LPS treatment in either LEW or PVG were identified with ≥1.5 fold change, and 85 transcripts intersected with those found in in 2-way ANOVA (Figure 1A). The transcripts that intersected from these three analyses accounted for one-third of the selected transcripts that were differentially expressed between the LEW and PVG animals (62/191) but accounted for more than two-thirds of the differentially expressed transcripts that were identified by comparing the LPS treatment groups (62/85). The disparate ratios indicated that the differentially expressed genes identified in the ASCs were mainly due to inherent differences between the LEW and PVG strains and partially due to the LPS stimulation.

Next, we compiled the three gene lists to form the union set of 358 differentially expressed genes. This set of genes was examined with pathway analysis using Agilent Genespring GX. We found 7 biological networks. The most important network, which was consistent with the network found when performing enrichment analysis of the 62 intersecting genes, was associated with 16 genes and a p-value of 1.855×10^−26^ (Figure 1B). The 16-gene subset associated with the top network was then clustered hierarchically into two groups on the basis of their normalized expression intensities. This analysis was performed by examining the differences among rat strains and treatment conditions with the average linkage option (Figure 1C). The 9 genes of cluster 1 were relatively highly expressed in ASCs of PVG rats and were not obviously altered with LPS treatment. In contrast, the 7 genes of cluster 2 were upregulated in response to LPS stimulation (Figure 1D). All 16 genes were grouped under different biological processes with significantly enriched functional categories using the GO analysis tool in Genespring GX. Table 1A and 1B are lists of significantly (p-value <0.05) overrepresented biological processes of the 2 clusters. The set of annotated genes within the indicated GO term and the p-value of these genes in the dataset are shown. The relative expression levels of each gene in each condition are also reported in the table. The GO terms involved with the developmental process and cell differentiation were significantly enriched in cluster 1, whereas GO terms involved with the response to external stimuli and cell differentiation were significantly enriched in cluster 2. This result indicated that a significant overrepresentation of cell differentiation processes among the functions of the 16 top network genes exists. We next examined the direct relationships between the 16 genes. Two significant transcription factors, GATA2 and NFATC4, individually clustered together in independent clusters within the cell differentiation function. This result suggested that the ASCs isolated from rats with acute rejection (LEW) and those with potential tolerance (PVG) mount different responses. The finding also implied that pivotal trans-activating events could be a result of both inherent differences and external immunological stimulation.

**Table 1:** The significantly associated Gene Ontology (biological process) terms overrepresented (corrected *p*<0.05) in the gene set of clusters.

A. Overrepresentation of cluster 1 genes					
Subset	GO ID	Term	Associated Genes	*p*-value	
**Cluster 1**					
	GO:0032502	Developmental Process	EDN1, ACTG2, TGFB3, GATA2, WT1, TGFB2, DES		0.001
	GO:0030154	Cell Differentiation	EDN1, GATA2, WT1, TGFB2, FHL2, COL4A1		0.005

Gene Symbol*	Fold change (compare to LEW control)				Description
	LEW		PVG		
	-	+LPS	-	+LPS	
FHL2	1	0.796	2.542	1.621	Rattus norvegicus four and a half LIM domains 2
COL4A1	1	1.266	4.138	3.224	Rattus norvegicus procollagen, type IV, alpha 1
EDN1	1	1.002	2.832	2.208	Rattus norvegicus endothelin 1
ACTG2	1	0.685	26.834	18.625	Rattus norvegicus actin, gamma 2, smooth muscle
TGFB3	1	0.468	5.456	3.643	Rattus norvegicus transforming growth factor, beta 3
GATA2	1	0.975	2.763	2.710	Rattus norvegicus GATA binding protein 2
WT1	1	0.873	2.710	2.472	Rattus norvegicus Wilms tumor 1
TGFB2	1	0.543	3.058	1.964	Rattus norvegicus transforming growth factor, beta 2
DES	1	1.008	15.947	10.759	Rattus norvegicus desmin

B. Overrepresentation of cluster 2 genes						
Subset	GO ID	Term	Associated Genes	*p*-value		
**Cluster 2**						
	GO:0009605	Response to External Stimulus	ADM, CXCL1, BDKRB1, CCL2		0.004	
	GO:0030154	Cell Differentiation	ADM. CCL2, CXCL5, NFATC4, SHC1		0.026	

Gene Symbol*	Fold change (compare to LEW control)				Description	
	LEW		PVG			
	-	+LPS	-	+LPS		
CXCL5	1	5.190	0.854	1.849	Rattus norvegicus chemokine (C-X-C motif) ligand 5	
NFATC4	1	1.790	0.767	1.142	Rattus norvegicus nuclear factor of activated T-cells, cytoplasmic, calcineurin-dependent 4	
SHC1	1	1.423	0.412	0.440	Rattus norvegicus src homology 2 domain-containing transforming protein C1	
ADM	1	1.314	0.294	0.508	Rattus norvegicus adrenomedullin	
CXCL1	1	21.601	0.735	15.803	Rattus norvegicus chemokine (C-X-C motif) ligand 1	
BDKRB1	1	1.931	1.017	2.402	Rattus norvegicus bradykinin receptor, beta 1	
CCL2	1	2.417	0.233	2.052	Rattus norvegicus chemokine (C-C motif) ligand 2	

* Genes that clustered together and occurred within the same GO terms.

Furthermore, 33 of the 625 differentially expressed genes in either the LEW or PVG groups with a ≥1.5 fold change after LPS stimulation were immune-related genes. These genes were classified as having functions related to the “cytokine activity” Molecular Function and the “inflammatory response” Biological Process (p-value  = 1.89×10^−4^) using the GO analysis tool in Genespring GX. As shown in Table 2, 4 of the 33 transcripts were associated with the acute inflammatory response (p-value  = 2.58×10^−5^). These genes in particular were upregulated and much higher in the LEW group upon LPS stimulation. Additionally, 4 of the 7 transcripts associated with inflammatory response as well as G-protein-coupled receptor activity were upregulated in the control ASCs of the LEW group than in those of the PVG group (underlined genes). The remaining 3 genes were downregulated in the ASCs of the LEW group including the chemokine ligand 12 with C-X-C motif (CXCL12) gene or named the stromal cell-derived factor-1 (SDF-1α). The expression results were successfully validated using quantitative RT-PCR (see Figure S1A), and indicated that immune-related G-protein-coupled receptors and cytokine activity were involved in the differentially regulated functions. Finally, 4 growth factors associated with angiogenesis of inflammatory responses (p-value  = 2.56×10^−5^) which expressed lower in the control ASCs of the LEW group than in those of the PVG group were also observed in the functional analysis.

**Table 2:** The 16 out of 33 isolated gene transcripts with immune-related function which significantly overrepresented in the following GO terms (corrected *p*<0.01).

Gene Symbol	Fold change				Description
	LEW	PVG	LEW/PVG		
	LPS/C	LPS/C	C/C		
**Immune-related Genes**					
***Acute inflammatory response (p-value = 2.58E-5)***					
IL1A	3.299	1.612	0.882	Rattus norvegicus interleukin 1 alpha	
HP	2.023	1.277	1.107	Rattus norvegicus haptoglobin	
CEBPB	2.364	1.628	0.991	Rattus norvegicus CCAAT/enhancer binding protein (C/EBP), beta	
IL6	3.161	1.220	1.233	Rattus norvegicus interleukin 6	
***G-protein-coupled receptor activity (p-value = 1.13E-8)***					
CCL11	1.294	1.734	2.391*	Rattus norvegicus chemokine (C-C motif) ligand 11	
CCL20	12.681	23.702	3.743*	Rattus norvegicus chemokine (C-C motif) ligand 20	
CCL7	3.022	5.351	3.076*	Rattus norvegicus chemokine (C-C motif) ligand 7	
CCL2	2.417	8.798	4.286*	Rattus norvegicus chemokine (C-C motif) ligand 2	
CXCL12	1.077	1.834	0.634*	Rattus norvegicus chemokine (C-X-C motif) ligand 12	
CX3CL1	1.170	1,628	0.798*	Rattus norvegicus chemokine (C-X3-C motif) ligand 1	
PF4	1.041	1.657	0.871*	Rattus norvegicus platelet factor 4	
***Angiogenesis (p-value = 2.56E-5)***					
IL1B	1.624	2.714	1.056	Rattus norvegicus interleukin 1 beta	
PGF	0.574	0.775	0.827	Rattus norvegicus placental growth factor	
TGFB2	0.543	0.643	0.327	Rattus norvegicus transforming growth factor, beta 2	
VEGFC	0.705	0.548	0.418	Rattus norvegicus vascular endothelial growth factor C	
PDGFA	0.659	0.681	0.759	Rattus norvegicus platelet derived growth factor, alpha	

Underlined genes represent those were expressed significantly higher in control LEW in array, and the 7 G-protein-coupled receptor genes were validated by quantitative RT-PCR (bolded number marked with a *).

### Knowledge-based enrichment analysis of overrepresented GO terms for the annotated miRNA transcripts

**Figure 2:**
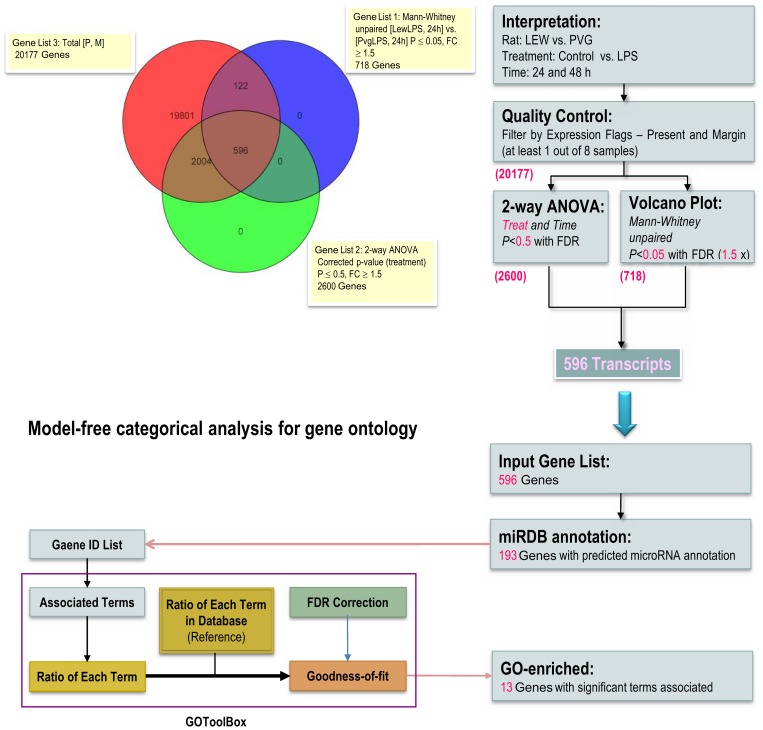
The less-stringent procedure of the knowledge-based enrichment analysis for detecting differentially expressed genes in the ASCs from the LEW and PVG rats. A Venn diagram showing the number of gene transcripts that were shared and distinct among the differentially expressed genes identified using the less-stringent statistical criteria. The number of annotated gene transcripts resulting from each step is given in purple. The application of the model-free categorical analysis for GO enrichment was performed using the online GOToolBox software.

The differential expression between the LEW and PVG with and without LPS stimulation was analyzed by calculating a statistic for each transcript with a p-value and controlling for the family-wise error rate (FWER) or the false discovery rate (FDR). These steps were necessary to account for the multiple comparisons performed via permutation or parametric distribution analysis. The selected genes were then hierarchically clustered on the basis of their intensities and experimental conditions. However, many genes that failed to meet the stringent criteria for significance are individually considered as not being differentially expressed. Their contribution to important effects on pathways and specific functional terms may be missed and this limitation was considered as a big challenge to find meaningful biological meanings. Thus, we applied profiling analyses with less stringent criteria and identified 596 differentially expressed transcripts between the LEW and PVG rats. These genes were calculated using a 2-way ANOVA with LPS condition and treatment durations as variables. We applied the Benjamini-Hochberg multiple testing correction and a p-value threshold of <0.5. We identified the genes in this list with those that were also differentially expressed and exhibited ≥1.5 fold changes in expression identified by the Mann-Whitney unpaired test. The Benjamini-Hochberg multiple testing correction and a p-value threshold of <0.5 were also applied to this analysis (Figure 2).

Moreover, 193 of the 596 unique genes were functionally annotated in the miRDB and were subjected to further analysis by the online GO Tool Box. There were 13 genes that were significantly enriched with overrepresented GO terms as shown in Table 3 (p-value <0.01). The table depicts the total number of genes annotated as functioning within the GO term and the associated p-value of each GO term. For genes that were upregulated in the ASCs of the PVG group, the GO terms related to cell cycle, cytokine production and metal ion binding were significantly over-represented (p-value <0.01). The same analysis applied to the genes upregulated in the ASCs of the LEW group identified Go terms significantly associated with extracellular region and endopeptidase activity. This result indicated that the 13 genes were predominantly involved in proliferation and cytokine activation with contact-dependent signaling.

**Table 3:** Functional classification of gene transcripts significantly enriched with over-represented GO terms (corrected *p*<0.05).

Gene Symbol	Fold change (compare to LEW control)					Description
	LEW		PVG			
	-	+LPS	-	+LPS		
**LEW > PVG**						
***Extracellular Region***						
PRL8A9	1	1.466	0.611	0.910	Prolactin family 8, subfamily a, member 9	
VEGFA	1	1.408	0.821	0.772	Vascular endothelial growth factor A	
COL12A1	1	1.014	0.369	0.326	Procollagen, type XII, alpha 1	
***Endopeptidase Activity***						
CASP6	1	1.141	0.647	0.755	Caspase 6	
**PVG > LEW**						
***Cell Cycle***						
TGFB2	1	0.543	3.058	1.964	Transforming growth factor, beta 2	
GADD45A*	1	0.451	0.378	0.247	Growth arrest and DNA-damage-inducible 45 alpha	
WT1	1	0.873	2.710	2.472	Wilms tumor 1	
***Metal Ion Binding***						
FHL1	1	0.712	2.572	1.274	Four and a half LIM domains 1	
ITPR1	1	0.569	2.272	1.130	Inositol 1,4,5-triphosphate receptor 1	
PLOD2	1	1.617	2.244	2.561	Procollagen lysine, 2-oxoglutarate 5-dioxygenase 2	
***Cytokine Production***						
IL1RL1	1	0.771	2.846	1.473	Interleukin 1 receptor-like 1	
IRF1	1	0.898	1.410	1.621	Interferon regulatory factor 1	
F2R	1	0.871	1.705	1.423	Coagulation factor II (thrombin) receptor	

The over-represented GO terms and associated genes were identified from the 193 out of 596 less stringent differentially expressed genes, which has been annotated in curated miRNA database.

### Promoter analyses and miRNA-targeting annotation of associated TFs

The above 13 genes within the same clusters with different GO functional terms were subjected to further promoter analysis using the PRIMA program integrated with the EXPANDER package. The 4 genes from the “extracellular region and endopeptidase activity” GO terms and 9 genes from the “cell cycle”, “metal ion binding” and “cytokine production” GO terms were analyzed. These genes were separately examined for the identification of putative transcription factors (TFs) whose binding sites were enriched in the promoters of the given gene sets. The region that was 3 kb upstream of the 5′-flanking sequence for each of the genes up-regulated in the LEW or PVG groups were analyzed (Table 4). The TF-binding site analysis identified 4 significant TFs, including PPARG, HOXA5, NFE2L2 and SREBP1. These TFs were consistently observed as regulators of the 596 genes differentially expressed between the LEW and PVG groups with and without LPS stimulation. Furthermore, 6 TFs, including EGR1, NFE2L2, GATA2, MYCN, NFATC4 (for NF-AT binding) and NKX2-5 were identified as promoter signatures of TF-binding motifs. These TFs were enriched within the genes of the PVG group's upregulated cluster and were also observed in the differentially expressed genes identified in the LEW and PVG datasets. Table 5 shows that all identified TF genes were significantly associated with 2 functional terms. They included “cell differentiation” (7 genes) and “ER-nucleus signaling pathway” (2 genes) GO categories. Interestingly, the 7 TF genes enriched in the “cell differentiation” category were also found in the same category with the 102 transcripts that were overrepresented in the 596 genes that were differentially expressed between the LEW and PVG groups. The differential expression of these 9 TF genes was confirmed using quantitative RT-PCR analysis (see Figure S1B). The validated fold changes are indicated by the bolded numbers in Table 5.

**Table 4:** Promoter enrichment analysis for overrepresentation of conserved TF binding sites within the LEW-upregulated and PVG-upregulated clusters.

Cluster	Transcription Factor (TF)	TRANSFAC ID	*p*-value*	Enrichment Factor**
***LEW up-regulated genes***				
	PPARG	M00515	0.005	10.96
	RXRA	M00631	0.009	7.786
	NFE2L2	M00821	0.025	4.939
	SREBP1	M00749	0.029	4.672
***PVG up-regulated genes***				
	NF-AT	M00935	0.001	7.526
	EGR1	M00243	0.008	4.280
	NFE2L2	M00821	0.012	6.934
	GATA2	M00076	0.019	8.363
	MYCN	M00055	0.025	5.632
	NKX2-5	M00240	0.037	3.987

The 9 TFs whose binding site profiles were significantly enriched in the 13 genes described in Table 3as well as observed in the 596 selected genes with less stringent criteria are presented.

* P-value indicated the significance of TF motif enrichment in the gene set relative to that in the background.

** Enrichment factor values represent the frequency of the TF motif in a gene set divided by its frequency in the background set and were calculated by PRIMA program.

**Table 5:** GO terms in Molecular Function overrepresentated in the enriched TF genes.

Subset	GO Term	p-value*	Transcripts
***9 Promoter-enriched TFs***			
	Cell Differentiation	6.02E-04	PPARG (**×2.68**), RXRA (**×1.57**), NFATC4 (**×0.63**), EGR1 (**×2.18**), GATA2 (**×3.59**), MYCN (**×2.35**), NKX2-5 (**1.21**)
	ER-nucleus signaling pathway	9.22E-03	SREBF1 (**×1.75**), NFE2L2 (**×2.97**)
***596 differentially expressed genes***			
	Cell Differentiation	7.87E-16	ACTA1, ADA, ADAM17, ADM, ALS2, ALX1, ANGPTL4, ANKRD1, ANPEP, ARHGAP24, AVPR1A, BMP6, BNIP3, BPGM, C1S, CAPN2, CHRD, CITED2, CLIC4, COL4A1, CRYAB, CTHRC1, CXCL5, DLX5, EDN1, **EGR1** *, EIF2B1, EIF2B3, F11R, F2R, FGF2, FHL1, FST, FSTL3, FYN, **GATA2**, GATA6, GJA1, GREM1, HOXB7, ID1, IL15, IRF1, ITGA1, ITGB1, JAK2, KLF4, LAMA5, LGALS3, LOC360228, LRRK2, MDK, MGP, MGST1, **MYCN**, MYH10, MYH11, NAB1, NBL1, NBN, NDRG2, NES, **NFATC4**, **NKX2-5**, PDGFRA, PDLIM5, PDLIM7, PGF, PHGDH, PICALM, PLA2G2A, PLCG2, PPARG, PRPF19, PSAP, RARRES2, RND1, RXRA, SELENBP1, SFRP2, SHC1, SLC1A3, SMAD4, SMARCD3, STAR, STAT5B, TACC1, TCF21, TCF4, TGFB1, TGFB2, THBS4, TMEM204, TNMD, TRPV4, TWIST2, UNC5B, VAMP5, VEGFA, VEGFC, WT1, ZFP36L1

Functional classification is shown for 104 gene transcripts represented as differentially expressed between ASCs of LEW and PVG. The average changes (fold change) validated by quantitative RT-PCR are given in parentheses.

* Bolded genes in the 596 gene set represent those were found in the motif-enriched TF genes.

Moreover, we performed *in silico* miRNA target prediction using the TargetScan and miRanda algorithms to identify the putative miRNAs that are predicted to target the 9 TF genes. The TF-targeting miRNAs and the number of targeted TF genes predicted by TargetScan or miRanda are provided in Table 6. Of the 16 candidate miRNAs that were predicted by the two algorithms, miR-27 was the most significantly correlated miRNA with cell differentiation functions as annotated by the FAME algorithm. This miRNA was also associated with the 5 TF targets (GATA2, RARA, PPARG, NFE2L2 and MYCN). Thus, the putative TF-targeting miR-27 that has been associated with adipogenesis and lipid metabolism was the first potential miRNA to be identified as being differentially regulated in ASCs from rat strains with distinct immune reactivity.

**Table 6:** Putative miRNAs associated with the 9 enriched TFs were predicted by TargetScan 5.1 and miRANDA software.

miRNA	No. of target TF (TargetScan 5.1)	No. of target TF (miRANDA)	No. Sum	*Significantly associated function by FAME		Associated TFs
				Term	*p*-value	
miR-27a/miR-27b	5	5	10	Cell differentiation	0.0055	GATA2, RXRA, PPARG, NFE2L2, MYCN
miR-144	3	4	7	Regulation of transcription, DNA-dependent	0.0026	GATA2, NFE2L2, MYCN
miR-128	3	3	6	Positive regulation of transcription, DNA-dependent	0.0025	GATA2, RXRA, NKX2-5
miR-101a/miR-101b	1	4	5	-		-
miR-200a/miR-200b/miR-200c	2	2	4	-		-
miR-124	3	1	4	-		-
miR-103	1	2	3	-		-
miR-106b	1	2	3	-		-
miR-132	1	2	3	-		-
miR-153	1	2	3	-		-
miR-17-5p	1	2	3	-		-
miR-193	1	2	3	-		-
miR-25	1	2	3	-		-
miR-340-5p	1	2	3	-		-
miR-34a/miR-34c	1	2	3	-		-
miR-29a/miR-29b/miR-29c	2	1	3	-		-

* The gene group of specific GO term is identified for each putative miRNA using the Functional Assignment of MiRNAs via Enrichment computational target prediction tool (FAME) in order to automatically infer the function affected by miRNAs. The significantly associated terms could be identified in the three top-ranked candidate miRNA genes.

### Differential expression of miR-27b and its responsiveness to LPS between the ASCs isolated from the LEW and PVG recipients after OLT

**Figure 3:**
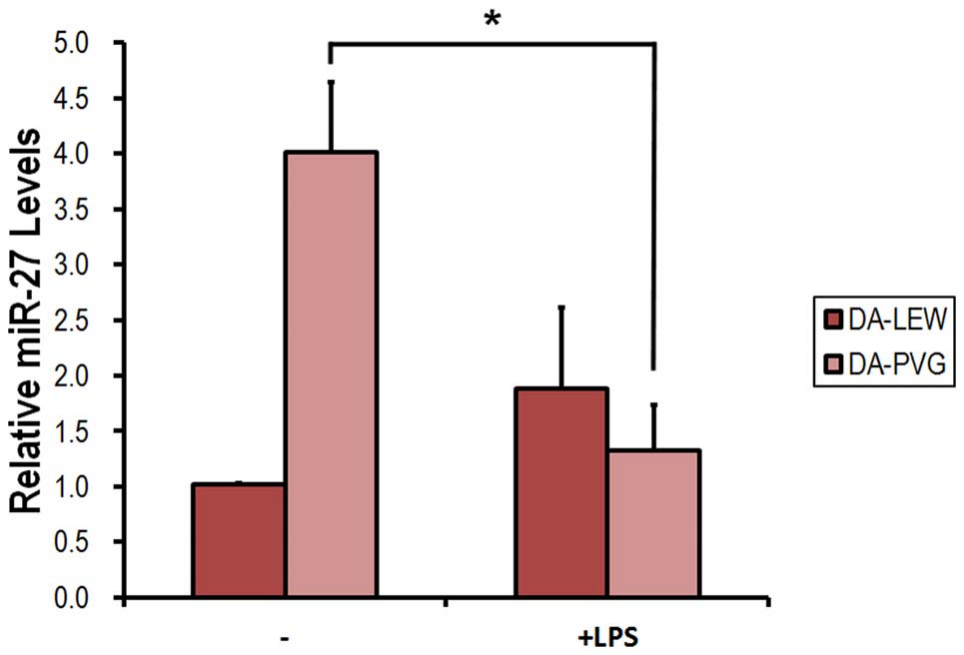
miR-27b expression in response to LPS treatment in the ASCs isolated from the LEW and PVG recipients after orthotopic liver transplantation (OLT). Isolated ASCs from the LEW and PVG recipients after OLT were incubated with 200 ng/ml LPS for 6 or 24 h. Total RNA was purified from the respective cell pellets, and the expression of miR-27b was analyzed by quantitative RT-PCR. The miRNA expression was normalized with U6. All results are expressed as the mean ± SD from three independent experiments. *p<0.05 compared with untreated ASCs.

In our orthotopic liver transplantation (OLT) models, rejection occurs during the early period after OLT (7 days after the operation). However, only the DA-PVG model naturally overcomes rejection without any immunosuppression. ASCs have been known to possess the capacity to inhibit the proliferation of immune cells upon allogeneic activation [14]. The immunosuppressive capacity of the MSC subtypes could be the key to applying these cells for therapy and in considering their therapeutic potency. This capacity also makes them interesting candidates for cell-based treatment of diseases and organ transplantation. To explore the potential role that regulates the immunosuppressive function of ASCs, we measured the expression of miR-27b in ASCs isolated from the recipient rats of the DA-LEW and DA-PVG OLT models. Strikingly, quantitative RT-PCR analysis showed that miR-27b was expressed at much higher levels in the ASCs isolated from the recipients of tolerance model compared with those isolated from the recipients of the acute rejection model. This result was gleaned from 3 independent pairs of OLT experiments (Figure 3). Moreover, we found that miR-27b levels were differentially regulated in the ASCs that were subjected to LPS stimulation. As shown in Figure 3, miR-27b was drastically down-regulated in the ASCs isolated from the DA-PVG recipients, but was expressed less and not significantly affected in the ASCs isolated from the DA-LEW recipients after LPS treatment.

### The effect of the miR-27b antagomir on the suppression of allogeneic CD4^+^ T cell proliferation observed in a co-culture with ASCs

**Figure 4:**
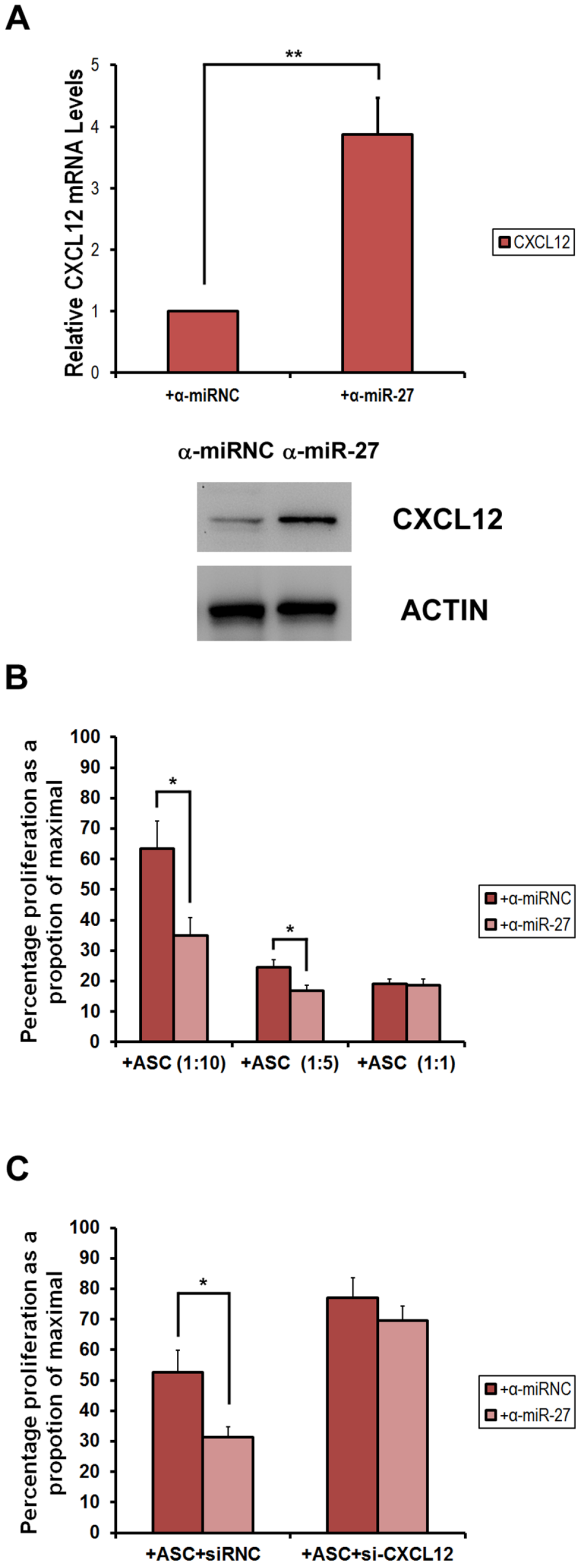
miR-27b regulates ASC-mediated suppression of CFSE-labeled allogeneic CD4^+^ T cell proliferation. (A) ASCs from Lewis rats were transfected with α-miR-27b or α-miRNC (25 µg/ml) for 24 h. Cells were harvested and the relative expression levels of CXCL12 mRNA were measured. Western blot analysis was also performed for CXCL12 and actin (as internal control). (B) A total of 2.5×10^−4^ purified splenic T cells were labeled with Carboxyfluorescein succinimidyl ester (CFSE). The cells were then cultured with Con A (1 µg/ml) (as 100% proliferation) and Con A + with different ratios of ASCs (1∶10, 1∶5, 1∶1, ASCs:T cells) with α-miR-27b or α-miRNC transfection. The cells were then stained with the anti-CD4 antibody, and proliferation was analyzed by FACS. A decrease in CFSE staining is an indication of proliferation. We quantified the percentage of CD4^+^ cells that have low levels of CFSE labeling. (C) Knocking out CXCL12 by siRNA transfection significantly impaired the suppressive actions of ASCs on T cell proliferation. Data are expressed as the mean ± SD of three independent experiments. *p<0.05; **p<0.01 compared with untreated ASCs.

To investigate the effect of miR-27b expression on the immunosuppressive activity mediated by ASCs, we determined the effect of the miR-27b antagomir (α-miR-27b) on the ASCs that can suppress the allogeneic proliferation of stimulated CD4^+^ T cells. We primarily examined the induction of CXCL12 mRNA and proteins in α-miR-27b-transfected ASCs comparing to the antagomir negative control (α-miRNC) (Fig. 4A). Furthermore, highly purified CD4^+^ T cells were CFSE-labeled, activated and cultured in the presence of mitomycin C-treated ASCs at different ratios. As shown in Fig. 4B, the proliferation of CFSE-labeled CD4^+^ T cells was significantly inhibited in the presence of ASCs. The impairment of CD4^+^ T cells were induced by miR-27b knockdown comparing to the antagomir negative control. To investigate whether CXCL12 affects the immune-modulatory capacity of ASCs, we then examined the role of ASC-derived CXCL12 in modulating the ASC-mediated suppression of CD4^+^ T cell proliferation. CXCL12 has been shown to exert its function through binding to its cognate receptor, CXCR4. This gene is constitutively expressed in ASCs [15]. We used siRNA to knock down CXCL12 expression, and then examined the effect of the knockdown on the suppression capacity of the ASCs. CXCL12 siRNA decreased the ASC production of CXCL12 by more than 90%, whereas the siRNA negative control (siRNC) did not affect the levels of ASC-derived CXCL12. As shown in Fig. 4C, the knockdown of ASC-derived CXCL12 by its specific siRNA significantly decreased the suppression of activated CD4^+^ T cell proliferation by α-miR-27b-transfected ASCs. These results further indicate that CXCL12 signaling is necessary for the ASC-mediated suppression of T cell proliferation that acts through miR-27b regulation.

## Discussion

There is a growing body of evidence on the immunosuppressive activity of MSCs demonstrating that MSCs of different origins suppress allo-activated immune cells [16]–[19]. Studies of the systemic gene expression changes that promote MSC-mediated immune modulation via microarray analysis are rare. However, these analyses would provide insight into new ways to achieve immunosuppression for transplants while MSCs are considered for clinical application. In this study, we proposed that specific intracellular signaling pathways modulate the gene expression important for the immunosuppressive properties of ASCs. These mechanisms are achieved through the regulation of TFs and TF-targeting miRNA interactions. We examined the differences in the LPS-induced mRNA expression response of ASCs from the acute rejection and spontaneous tolerance groups. We aimed to identify the genes that were differentially expressed between these groups and the significantly associated GO functional terms in integrating miRNA-targeted information. Our multi-step computational strategy for systematically predicting the TF-targeted miRNAs involved in the immunosuppressive function of the ASCs was based on enrichment analysis of miRNA annotations and functional terms. Hierarchical clustering demonstrated that the differences observed in gene expression accounted for the genetic inheritance between the LEW and PVG groups was greater than those accounted for by the LPS exposure. The data consistency could be proved between the high stringency (358 genes) and less stringency (596 genes) criteria in Figure 1 and Figure 2, respectively, where the top-ranked gene group of pathway analysis for the 596 genes also mapped on the same core in gene network of direct interactions (p-value  = 1.059×10^−20^, Figure S2). The extensive modulation of the differentially expressed genes in the two rat groups show that the inherent ASC expression differences is part of a constellation of regulatory mechanisms and is unlikely to stand alone. These patterns imply that potent miRNA-mediated regulation may be involved at the post-transcriptional level. Our approach efficiently identified miR-27 among the chosen putative miRNA targets for further validation. The differential expression of miR-27b in the ASCs was observed through comparing between the LEW and PVG rats, and the ratio of miR-27b between LEW and PVG is 1.439 (LEW/PVG, p<0.05) by qRT-PCR (Figure S3A). However, the detailed mechanisms of how miR-27b regulates the immunosuppressive function of ASCs would be studied and further analysis is necessary to investigate the effectors responsible for modulating the production of cytokines/chemokines, allogeneic induction of regulatory T cells and stem cell differentiation.

Furthermore, of the 625 genes that were differentially expressed in the ASCs of the LEW or PVG groups with and without LPS stimulation, 20 genes exhibited cytokine activity-related functions. Seven genes were involved in G-protein coupled chemokine activity (see Table S1). These included 4 inflammatory genes, CCL2 (MCP-1), CCL7, CCL11 and CCL20. These genes were expressed at higher levels in the naïve LEW group and in response to LPS stimulation. These results suggest that the naïve PVG rats have suppressed inflammatory responses in comparison to the LEW group. Interestingly, CXCL12 was differentially expressed between the LEW and PVG groups as well as between the LPS treatment groups. The chemokine ligand 12 has a C-X-C motif or named stromal cell-derived factor-1 alpha (SDF-1α), which is known to be involved in the regulation of migration, survival as well as the development of multiple types of stromal stem cells. Quantitative RT-PCR was performed to validate the higher expression levels of CXCL12 in the ASCs of the PVG group compared to the LEW group.

CXCL12 is a major chemotactic factor for stem cells [20]. It is also cardio-protective after myocardial infarction [21], [22]. Several reports have shown that CXCL12 has multiple effects on myocardial structure and function, including cardioprotection, myogenesis, angiogenesis and anti-inflammation [21], [23]–[26]. Overexpression of CXCL12 in MSCs can repair the infarcted heart through the effects of CXCL12-mediated nutrition on cardiocytes and promotion of angiogenesis [27], [28]. CXCL12 can also enhance the survival of MSCs via the activation of Akt [29], [30]. Furthermore, murine and human studies have shown that SDF-1 and its receptor CXCR4 participate in the regulation of stem/progenitor cell trafficking, which elicits the signals required for their homing and recruitment to injured liver [31]–[34]. Local SDF-1 injection can efficiently increase the homing of bone marrow-derived MSCs to sites of traumatic injury and improve wound healing [35]. The protective role of CXCL12 in diet-induced atherosclerosis has been discovered recently [36]. Moreover, CXCL12 secreted from transplanted MSCs have neuroprotective effects *in vivo* and *in vitro*. The cardiac stem cell-derived paracrine action of CXCL12 is cardioprotective in *ex vivo* studies. These data suggest that CXCL12/SDF-1 may play a critical protective role in the healing of injured tissues and organ regeneration [37], [38]. Here we found that CXCL12 mRNA and protein expression were highly induced by miR-27b knockdown in LEW ASCs, and this induction is responsible for the inhibition of CD4^+^ T-cell proliferation in vitro. This finding is consistent with that of Lu et al. (2012) who found that miR-27b could suppress SDF-1α protein expression with direct interaction with its 3′UTR [39]. The miR-27b knockdown in ASCs from naïve PVG also induced CXCL12 expression and repressed T-cell proliferation, and the level of induced CXCL12 as well as the suppression of T-cell proliferation was significantly higher than those in ASCs from LEW (Figure S3B, C). Furthermore, overexpression of miR-27b on ASCs from both LEW and PVG significantly de-repressed the T-cell proliferation in co-culturing with ASCs from both LEW and PVG, and the CXCL12 expression was brought to similar levels with no significant difference. The relative level of miR-27b is significantly less in ASCs of naïve PVG, however is 4-time higher in PVG after OLT and drastically decreased upon LPS stimulation (Figure 3). In our unpublished data, we also observed that miR-27b-transfected ASCs from the LEW group enhanced the inhibitory effect on CD4^+^ T cells under inflammatory conditions. The drastic down-regulation of miR-27b after LPS treatment was only observed in ASCs isolated from DA-PVG recipients but not DA-LEW. This implies that the expression levels of CXCL12 may be crucial for the survival of transplanted rats, and indicating that miR-27b may play an important role in modulating the immunosuppressive and protective paracrine effects of CXCL12 on ASCs as well as other MSCs. The mechanisms underlying these complicated responses need further investigation. The miRNAs are important regulators of putative repair and protective mechanisms that may be important for the development of MSC-based therapy. It has been proposed that the therapeutic modulation of functional miRNAs in MSCs may improve cell survival and therapeutic efficacy either by mimicking or antagonizing miRNA activity [40]. Using a knowledge-based approach, we are the first to demonstrate that increased expression of several TFs under inflammatory conditions and the relative expression of miR-27b in the ASCs of the PVG and LEW rat groups are important mechanisms that regulate the cell differentiation and immunosuppressive function of ASCs. We propose that the higher level of miR-27b accumulation in the ASCs of the PVG group compared with those of the LEW group and its down-regulation upon allogeneic stimulation after OLT precisely regulate target expression and contribute to the immunosuppressive activity of ASCs. This regulation is critical for preventing processes such as differentiation and apoptosis and improving responsiveness to T cells. Our results provide the first evidence that miR-27b exhibits a regulatory role on ASC-mediated T-cell proliferation inhibition. Detailed functional analysis of miR-27b effects in ASCs would provide further insights into the pivotal role of miRNAs in mesenchymal stem cells.

## Materials and Methods

### Animals and ethics

Male DA (major histocompatibility complex haplotype RT1^a^) and PVG (RT1^c^) rats were obtained from Japan SLC (Hamamatsu, Japan) and the Institute of Laboratory Animals of the Graduate School of Medicine, Kyoto University (Kyoto, Japan) respectively. Male LEW (RT1^l^) rats of four-week old were obtained from the National Laboratory Animal Breeding and Research Center (Taipei, Taiwan). All of the animals were maintained in specific pathogen-free animal facilities with water and commercial rat food provided ad libitum. Our experimental design was reviewed and approved by the Institutional Animal Care and Use Committee of the Kaohsiung Chang Gung Memorial Hospital (approval No: 2009122102), and the Committee recognizes that the proposed animal experiment follows the Animal Protection Law by the Council of Agriculture, Executive Yuan, R.O.C. and the guideline as shown in the Guide for the Care and Use of Laboratory Animals as promulgated by the Institute of Laboratory Animal Resources, National Research Council, USA.

### Expression profiling and data generation

The microarray experiment was designed to compare gene expression profiles of ASCs with and without 1 µg/ml LPS treatment among four groups, i.e., LEW-C, PVG-C, LEW-LPS and PVG-LPS for 24 and 48 h. We collected twenty-four samples and carried out eight microarray experiments with 3 pooled samples in each array. Purified RNA from ASCs by Trizol® reagent (Life Technologies, Inc., Gaithersburg, MD, USA) was quantified using a NanoDrop spectrophotometer (Rockland, DE, USA). RNA integrity was verified with a BioAnalyzer 2100. Then, high quality RNA (RIN >7) 200 ng was transcribed into cDNA with a double reverse transcriptase-PCR technique, and *in vitro* transcription was performed to generate biotin-labeled cDNA for subsequent hybridization on RatRef-12 Expression BeadChip® (Illumina, San Diego, CA, USA). This chip contains 22,523 transcripts and variants, including 22,228 well-characterized rat genes selected primarily from the NCBI RefSeq database (Release 16), and was used in accordance with the manufacturer's instructions. The BeadChip was scanned on a high resolution Illumina BeadArray reader, using a two-channel, 0.8 μm resolution confocal laser scanner. Finally, gene expression analysis software was used for data analyses of differential expression profiles (GeneSpring GX software, version 10, Agilent Technologies). Microarray data for all the ASC samples have been deposited in the Gene Expression Omnibus (GEO) database. They are accessible through the GEO series web accession number GSE41262.

### Gene ontology (GO) classification and promoter analysis

Genes that were identified to be differentially expressed between LEW and PVG and significantly altered by LPS were classified into GO categories over-represented in our dataset in the context of the whole genome reference using GO Tool Box [41] with Benjamini & Hochberg correction from the hypergeometric test.

It is thought that genes with strongly correlated mRNA expression profiles are more likely to be regulated via the same control mechanisms. To detect the *cis*-regulatory binding elements that control the expression of altered genes in the specific clusters, we applied EXPANDER software [42] for promoter analysis. 3 kb upstream regions from the transcription start site of such genes in a cluster were retrieved using Ensembl [43] and the transcription factor binding within this region was determined using PRIMA program in EXPANDER package that detects over-represented binding motifs of known TFs in the cluster relative to background (TF enrichment is indicated by p-value). The enrichment factor is the ratio between the prevalence of the TF hits in the clustered genes and in the background set of promoters.

### miRNA Target prediction and enrichment analysis

The targeting miRNAs for selected TF genes were predicted by two published algorithms and the data was retrieved from the public websites (TargetScan, http://targetscan.org; miRanda, http://microrna.snager.ac.uk). The number of targeted TF genes predicted by both algorithms was summarized for all the conserved targeting miRNAs. The correlation between each predicted miRNA and its associated targets was evaluated by over-representation analysis using the FAME algorithm of EXPANDER package [44]. An empirical enrichment p-value for each pair of predicted miRNA and the gene group was computed with its associated functional (GO) term.

### Isolation of adipose-derived mesenchymal stem cells (ASCs) from rat and human tissues

Eight- to 12- week-old female Lewis rats were used for the isolation of rat ASCs. Adipose tissue cells were isolated from rat inguinal and interscapular adipose tissues. Briefly, the adipose tissue was dissociated mechanically and digested at 37°C in HBSS buffer (Gibco) containing 1 mg/ml collagenase I (Sigma) for 20 min. After digestion, the contents were filtered with a 100 μm filter prior to centrifugation at 800× g for 10 min at room temperature. The cell pellet was resuspended in washing buffer (PBS with 1% FBS) and then centrifuged again at 800× g for 5 min at room temperature. After being resuspended again in washing buffer and filtered through a 25 μm filter, mature adipocytes were separated from the stromal fraction by centrifugation (800× g for 10 min) at room temperature. The pellet was resuspended in culture medium and an aliquot of the cell suspension was then seeded (10,000 cells/cm^2^) in Dulbecco's modified Eagle's medium (DMEM; Gibco) with 10% fetal bovine serum (FBS; Gibco) medium and maintained in a 5% CO_2_ and humidified atmosphere. Twenty-four hours after plating, all non-adherent cells were removed by changing the medium. Sub-confluent ASCs were obtained after 5 days.

### Preparation of primary CD4^+^ T cells

Using standard protocols, primary T cells were prepared from spleens that had been collected aseptically from euthanatized DA rats. For T lymphocyte cultures, single-cell suspensions were prepared by pushing the tissue pieces through a nylon mesh screen (BD Bioscience), washing once with phosphate-buffered saline (PBS) containing 0.5% bovine serum albumin (BSA), lysing erythrocytes by incubation in a lysis buffer (BioWhittaker) for 5 min, and washing again with PBS. The splenocytes were then incubated with anti-CD4 MicroBeads (Miltenyi Biotec, Germany) for 15 min at 4–8°C, washed, resuspended in 500 µl of buffer, and loaded onto the MACS columns for the positive selection of CD4^+^ T lymphocytes. To activate the rat lymphocytes, concanavalin A (ConA) (Sigma) was added at a final concentration of 10 mg/ml. After an overnight incubation, the cells were washed twice with PBS and then resuspended in fresh medium.

### Reagents and transient transfection with microRNA antagomir and small interfering RNA

The endotoxin (lipopolysaccharide, LPS) was purchased from Sigma (St Louis, MO, USA). miRNA antagomir was purchased from Ambion (Austin, TX, USA). The small interfering RNA (siRNA) for CXCL12 used in the present study were purchased from Sigma-Aldrich, Inc. ASCs were cultured in basal media consisting of DMEM supplemented with 2 mM L-glutamine and 10% FBS until the cells reached 60% confluency; cells were then transfected with the miR-27b antagomir (α-miR-27), the anti-miR negative control (α-miRNC, Ambion) or CXCL12 siRNA using TurboFect^TM^ siRNA Transfection Reagent (Fermentas Life Science) according to the manufacturer's protocol. Then, the medium was changed to fresh DMEM with 10% FBS, and the cells were incubated for another 48 h. The knockdown efficiency was assessed by measuring the mRNA expression levels. The efficiency of the CXCL12 knockdown was more than 95% as measured by mRNA levels.

### RNA isolation and quantitative RT-PCR

Quantitative RT-PCR was performed for validation of gene expression microarray data. Total RNA was isolated from the cells using the RNeasy kit from Qiagen (Valencia, CA, USA) according to the manufacturer's protocol. Reverse transcription was performed with 1 μg RNA using the First-Strand cDNA synthesis kit (Promega, Madison, WI, USA) or the miScript Reverse Transcription Kit (Qiagen) for the transcription of miRNA as described by the manufacturer. The quantitative RT-PCR reaction was performed on an ABI 7500 Fast Real-Time PCR System with the SDS 1.4 program using the ABI TaqMan Fast Universal PCR master mix or the TaqMan Universal PCR master mix for miRNA (Applied Biosystems, Foster, CA, USA). The primers and TaqMan MGB probes were obtained from Applied Biosystems, and the final concentration of primers and probes was 300 nM and 250 nM, respectively. The cycling profile for each run was 95°C for 20 seconds and 40 cycles of 95°C for 3 seconds followed by 60°C for 30 seconds using the default ramp rate. The normalization was performed with rat Glyceraldehyde 3-phosphate dehydrogenase (GAPDH) primers. For miRNA, the primers and TaqMan probes for miR-27b (P/N: 44279775, ID: 000409) and U6 snRNA (P/N: 44279775, ID: 001973) were obtained from Applied Biosystems. The cycling profile for each run was 50°C for 2 minutes, 95°C for 10 minutes and 40 cycles of 95°C for 15 seconds followed by 60°C for 1 minute using the default ramp rate. The normalization was performed with U6 snRNA primers. Comparative RT-PCR data including non-template controls was done in triplicate. The fold increase in the expression of cytokine mRNA was calculated with the comparative 2^−ΔΔCt^ method.

### Data analysis and statistics

The fluorescence intensities from array scans were averaged across all gene probes on each array using the Percentile shift algorithm to process and normalize raw data from microarray gene chips. For the normalization of raw data, all signal values were log transformed (log base 2), each measurement was divided by the 75.0th percentile of all measurements in that sample, and each gene was divided by the median of its measurements in all samples. The results from eight arrays were analyzed by Mann-Whitney un-paired test or 2-way ANOVA and a Benjamini and Hochberg false discovery rate (FDR) multiple gene correction was applied.

Each experiment of other analyses was performed three times, and the statistical significance of the data was calculated using Student's *t*-test. A value of p<0.05 was considered to indicate statistical significance.

## Supporting Information

Figure S1 **Quantitative validation of gene expression levels by RT-PCR.** (A) 7 selected genes from the significantly associated GO term “G protein-coupled receptor activity” with immune-related function, and (B) 9 TF genes identified from the promoter enrichment analysis successfully represented significant differences between ASCs of LEW and PVG by quantitative RT-PCR. The results (fold changes) are also shown in Table 5 by given in parentheses. *p<0.05 and **p<0.01 compared with untreated ASCs.
(TIF)

Figure S2 **The top interacting network analyzed among the shared genes identified by the comparisons with less stringent statistical criteria.** The functional interaction networks among the intersected 596 genes with less stringent criteria and showed in Fig. 2, were analyzed using the significant pathway analysis tool in Genespring GX. The top networks of significant “direct interactions” and their associated p-values are also indicated.
(TIF)

Figure S3 **Differential expression of miR-27b but not miR-27a between ASCs of LEW and PVG negatively regulated CXCL12 expression and the suppression of CD4^+^ T-cell proliferation.** (A) The expression of miR-27a and miR-27b were analyzed in ASCs from LEW and PVG by quantitative RT-PCR. Each miRNA expression was normalized with U6. All results are expressed as the mean ± SD from three independent experiments. *p<0.05 compared with LEW ASCs as control. (B) ASCs from LEW and PVG rats were transfected with α-miR-27b, α-miRNC or miR-27b-mimic (25 µg/ml) for 24 h. Cells were harvested and the relative expression levels of CXCL12 mRNA were measured. (C) A total of 2.5×10^−4^ purified splenic T cells were labeled with CFSE. The cells were then cultured with Con A (1 µg/ml) (as 100% proliferation) and Con A plus with LEW or PVG ASCs (1∶10, ASCs:T cells) with α-miR-27b, α-miRNC or miR-27b-mimic transfection. The cells were then stained with the anti-CD4 antibody, and proliferation was analyzed by FACS. A decrease in CFSE staining is an indication of proliferation. We quantified the percentage of CD4^+^ cells that have low levels of CFSE labeling. *p<0.05 and **p<0.01 compared with untreated ASCs.
(TIF)

Table S1 **TeqMan probe-based assays used in quantitative RT-PCR validation.**
(DOCX)
