# Supplementary figures and images for: Identification of miR-27b as a Novel Signature from the mRNA Profiles of Adipose-Derived Mesenchymal Stem Cells Involved in the Tolerogenic Response
Source: PLoS One. 2013 Apr 16;8(4):e60492. doi: 10.1371/journal.pone.0060492 (PMC3628792; doi:10.1371/journal.pone.0060492)

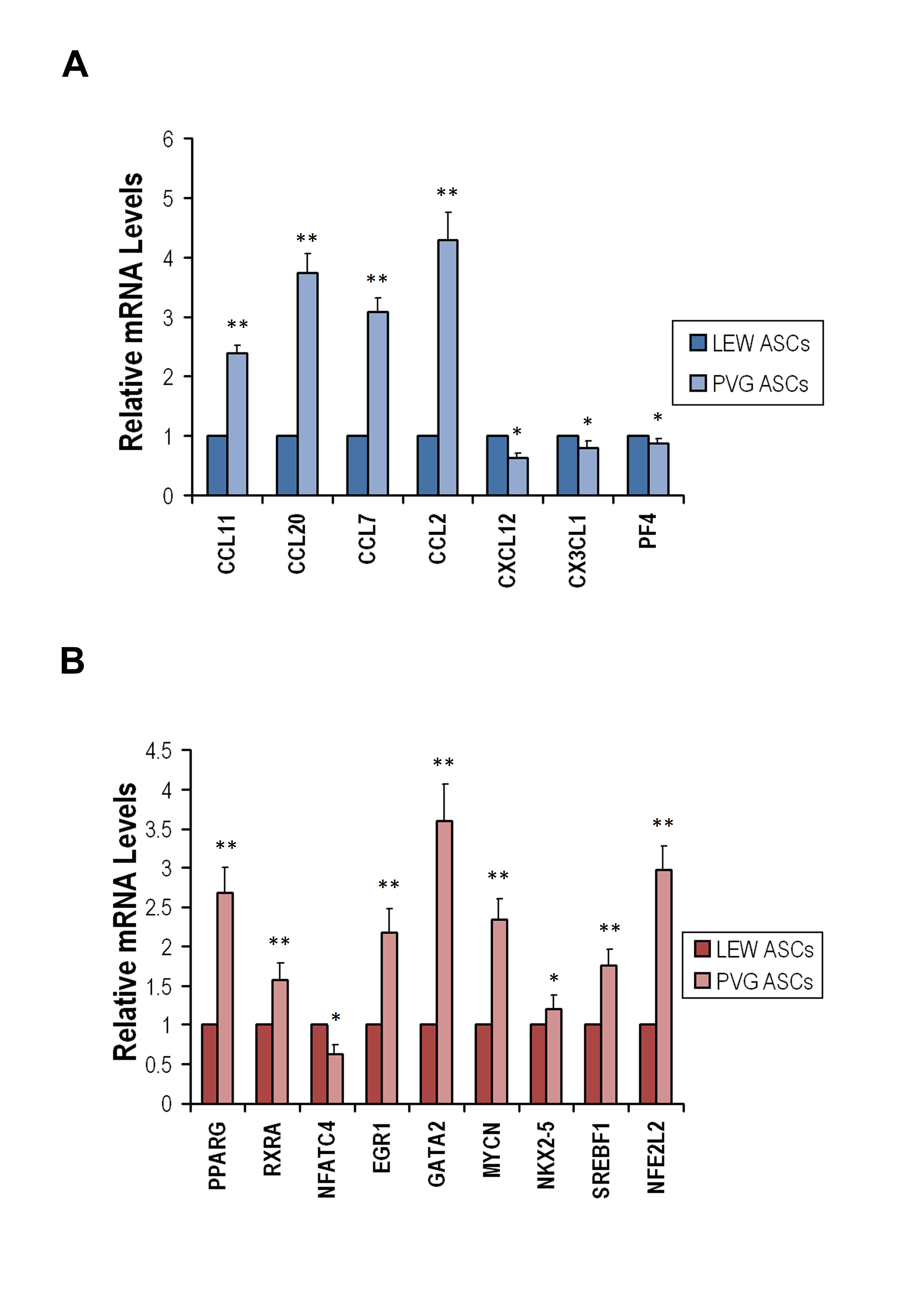

Supplement: Figure S1 — Quantitative validation of gene expression levels by RT-PCR. (A) 7 selected genes from the significantly associated GO term “G protein-coupled receptor activity” with immune-related function, and (B) 9 TF genes identified from the promoter enrichment analysis successfully represented significant differences between ASCs of LEW and PVG by quantitative RT-PCR. The results (fold changes) are also shown in Table 5 by given in parentheses. *p<0.05 and **p<0.01 compared with untreated ASCs. (TIF) [file pone.0060492.s001.tif]

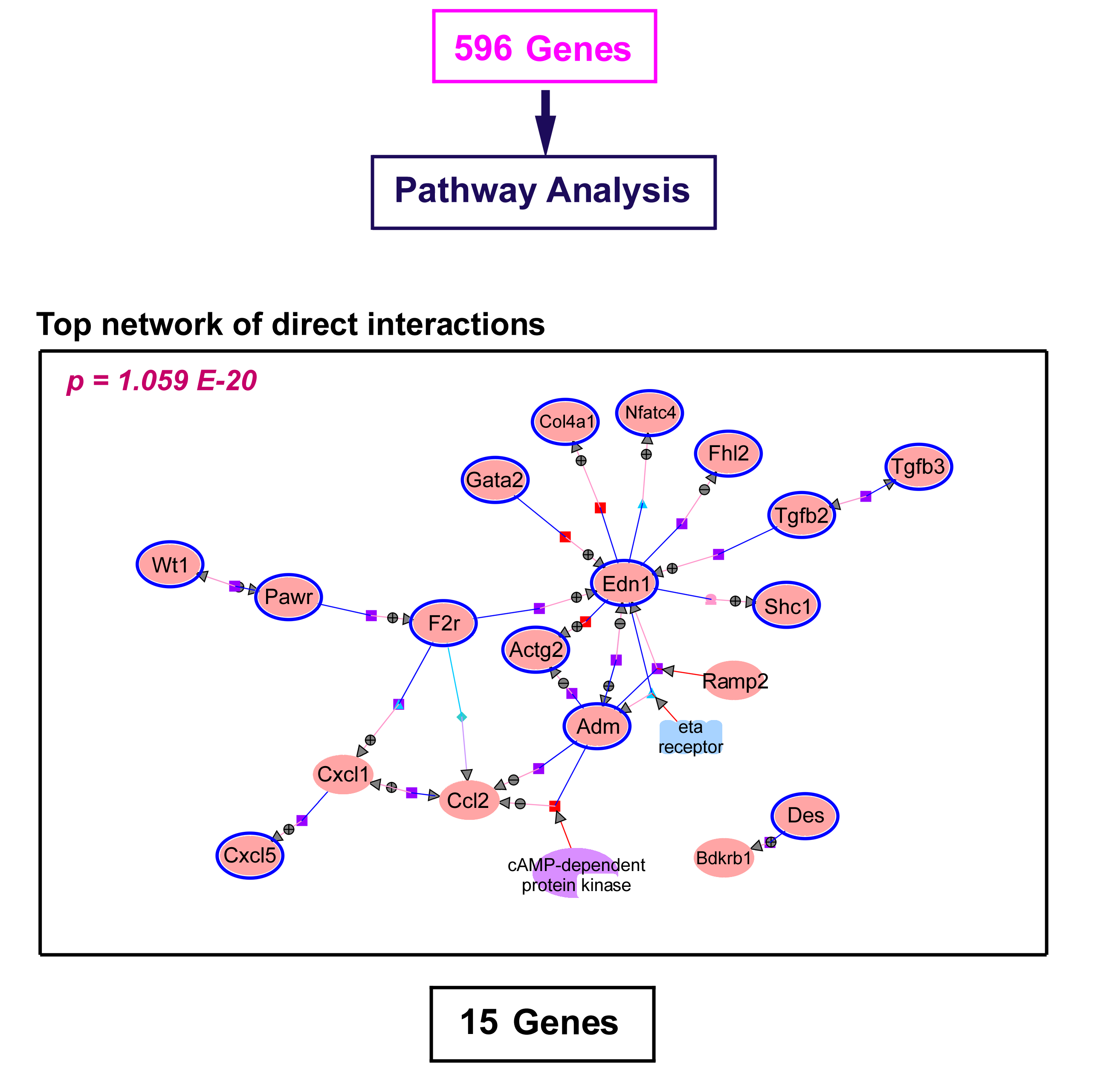

Supplement: Figure S2 — The top interacting network analyzed among the shared genes identified by the comparisons with less stringent statistical criteria. The functional interaction networks among the intersected 596 genes with less stringent criteria and showed in Fig. 2, were analyzed using the significant pathway analysis tool in Genespring GX. The top networks of significant “direct interactions” and their associated p-values are also indicated. (TIF) [file pone.0060492.s002.tif]

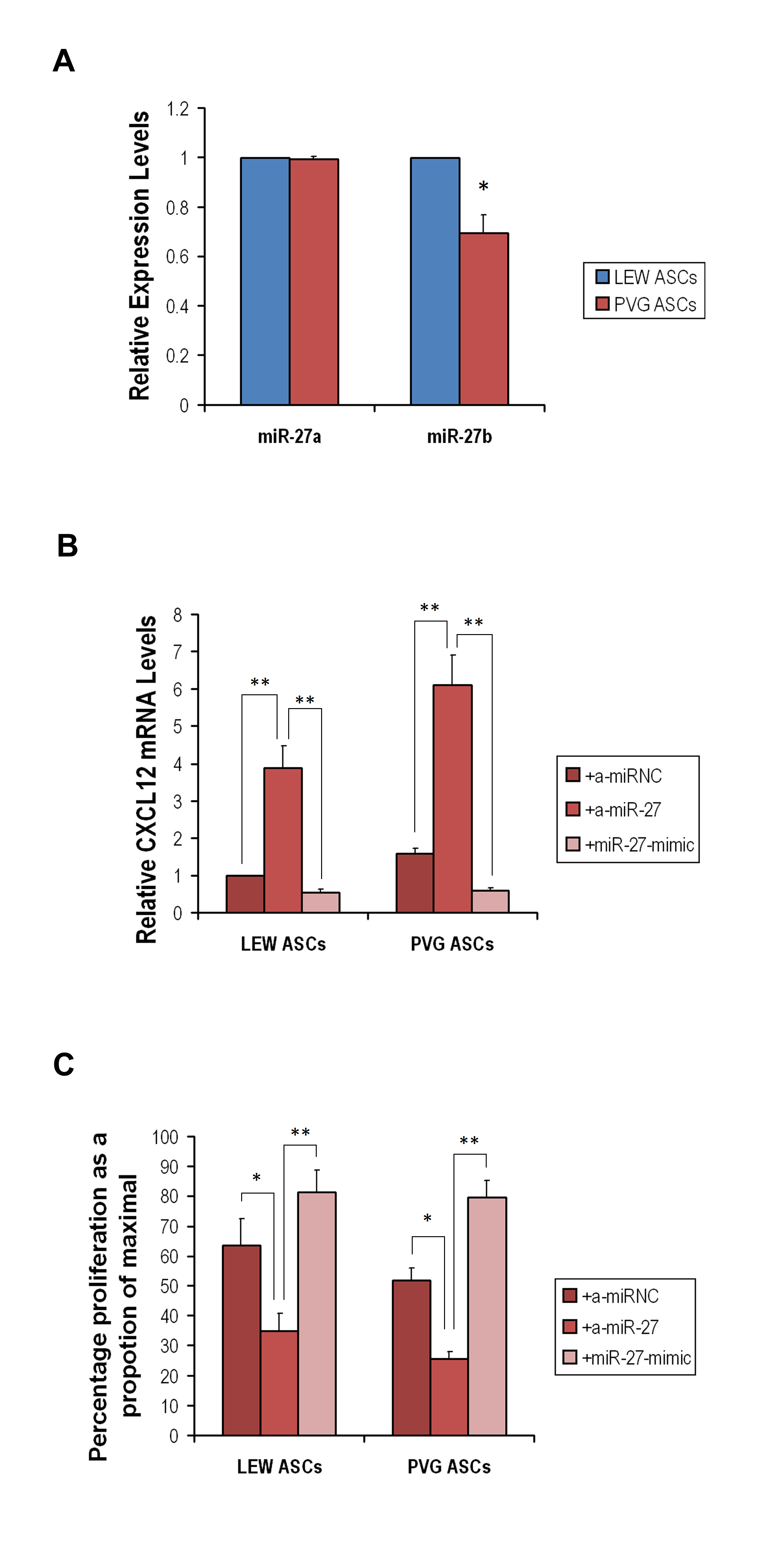

Supplement: Figure S3 — Differential expression of miR-27b but not miR-27a between ASCs of LEW and PVG negatively regulated CXCL12 expression and the suppression of CD4+ T-cell proliferation. (A) The expression of miR-27a and miR-27b were analyzed in ASCs from LEW and PVG by quantitative RT-PCR. Each miRNA expression was normalized with U6. All results are expressed as the mean ± SD from three independent experiments. *p<0.05 compared with LEW ASCs as control. (B) ASCs from LEW and PVG rats were transfected with α-miR-27b, α-miRNC or miR-27b-mimic (25 µg/ml) for 24 h. Cells were harvested and the relative expression levels of CXCL12 mRNA were measured. (C) A total of 2.5×10^−4^ purified splenic T cells were labeled with CFSE. The cells were then cultured with Con A (1 µg/ml) (as 100% proliferation) and Con A plus with LEW or PVG ASCs (1∶10, ASCs:T cells) with α-miR-27b, α-miRNC or miR-27b-mimic transfection. The cells were then stained with the anti-CD4 antibody, and proliferation was analyzed by FACS. A decrease in CFSE staining is an indication of proliferation. We quantified the percentage of CD4+ cells that have low levels of CFSE labeling. *p<0.05 and **p<0.01 compared with untreated ASCs. (TIF) [file pone.0060492.s003.tif]
