# Supplementary material for: Identification of miR-27b as a Novel Signature from the mRNA Profiles of Adipose-Derived Mesenchymal Stem Cells Involved in the Tolerogenic Response
Source: PLoS One. 2013 Apr 16;8(4):e60492. doi: 10.1371/journal.pone.0060492 (PMC3628792; doi:10.1371/journal.pone.0060492)
Supplement: Table S1 — TeqMan probe-based assays used in quantitative RT-PCR validation. (DOCX) [file pone.0060492.s004.docx]

**Table S1. TeqMan probe-based assays used in quantitative RT-PCR validation.**

| **Gene Symbol** | **Gene Expression Assay** | **Gene Name** |
| --- | --- | --- |
| CCL11 | Rn00569995_m1 | chemokine (C-C motif) ligand 11 |
| CCL20 | Rn00570287_m1 | chemokine (C-C motif) ligand 20 |
| CCL7 | Rn01467286_m1 | chemokine (C-C motif) ligand 7 |
| CCL2 | Rn00580555_m1 | chemokine (C-C motif) ligand 2 |
| CXCL12 | Rn00573260_m1 | chemokine (C-X-C motif) ligand 12 |
| CX3CL1 | Rn00593186_m1 | chemokine (C-X3-C motif) ligand 1 |
| PF4 | Rn01768297_g1 | platelet factor 4 |
| PPARG | Rn00440945_m1 | peroxisome proliferator-activated receptor gamma |
| RXRA | Rn00441185_m1 | retinoid X receptor, alpha |
| NFATC4 | Rn01488729_m1 | nuclear factor of activated T cells, cytoplasmic, calcineurin dependent 4 |
| EGR1 | Rn00561138_m1 | early growth response 1 |
| GATA2 | Rn00583735_m1 | GATA binding protein 2 |
| MYCN | Rn01473353_m1 | v-mycmyelocytomatosis viral related oncogene, neuroblastoma derived |
| NKX2-5 | Rn00586428_m1 | NK2 homeobox 5 |
| SREBF1 | Rn01495769_m1 | sterol regulatory element binding transcription factor 1 |
| NFE2L2 | Rn00477784_m1 | nuclear factor, erythroid derived 2, like 2 |
